# A Flexible and Lightweight Biomass-Reinforced Microwave Absorber

**DOI:** 10.1007/s40820-020-00461-x

**Published:** 2020-06-11

**Authors:** Yan Cheng, Justin Zhu Yeow Seow, Huanqin Zhao, Zhichuan J. Xu, Guangbin Ji

**Affiliations:** 1 College of Materials Science and Technology, Nanjing University of Aeronautics and Astronautics, Nanjing, 210016 People’s Republic of China; 2 School of Materials Science and Engineering, Nanyang Technological University, 50 Nanyang Avenue, Singapore, 639798 Singapore; 3 Singapore-HUJ Alliance for Research and Enterprise, NEW-CREATE Phase II, Campus for Research Excellence and Technological Enterprise (CREATE), Singapore, 138602 Singapore

**Keywords:** Flexible, Biomass, Microwave absorption, Dielectric loss, Magnetic loss

## Abstract

Developing a flexible, lightweight and effective electromagnetic (EM) absorber remains challenging despite being on increasing demand as more wearable devices and portable electronics are commercialized. Herein, we report a flexible and lightweight hybrid paper by a facile vacuum-filtration-induced self-assembly process, in which cotton-derived carbon fibers serve as flexible skeletons, compactly surrounded by other microwave-attenuating components (reduced graphene oxide and Fe_3_O_4_@C nanowires). Owing to its unique architecture and synergy of the three components, the as-prepared hybrid paper exhibits flexible and lightweight features as well as superb microwave absorption performance. Maximum absorption intensity with reflection loss as low as − 63 dB can be achieved, and its broadest frequency absorption bandwidth of 5.8 GHz almost covers the entire Ku band. Such a hybrid paper is promising to cope with ever-increasing EM interference. The work also paves the way to develop low-cost and flexible EM wave absorber from biomass through a facile method.

**Electronic supplementary material:**

The online version of this article (10.1007/s40820-020-00461-x) contains supplementary material, which is available to authorized users.

## Highlights

- A flexible and lightweight microwave absorber was prepared by a vacuum filtration method.
- The remarkable microwave absorbency makes the absorber paper attractive in wireless wearable electronics field.

**Electronic supplementary material:**

The online version of this article (10.1007/s40820-020-00461-x) contains supplementary material, which is available to authorized users.

## Graphical abstract

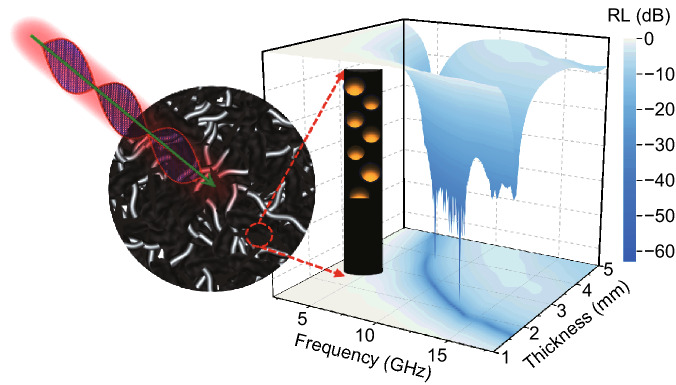

## Introduction

Wireless electronics, especially wearable electronics, have attracted growing public attention through recent technology advances. With increasing expectation on the efficiency of such electronics, sensitivity of these electronics to input signals needs to be improved while decreasing their susceptibility to unwanted environmental interference [1, 2]. A viable solution to serious signal interference from electronics nearby is proposed, in which an electromagnetic (EM) absorption layer is directly attached to the surface of an electronic device [3]. The EM absorption layer is able to transform the unwanted EM energy into thermal energy and/or other energy forms, protecting the electronic equipment from signal interference. An aspect from the EM wave absorption capability and flexibility is required in an EM absorber to allow it to adapt to the shape of wearable electronics [4]. Although traditional binder-containing microwave absorbers have made great progress toward high absorption efficiency, they are still hardly used practically because of their lack of stability and poor mechanical properties [5].

Recent progress demonstrates that mixing conductive filler (such as carbon nanotube (CNT) and graphene) with polymer to create a composite film is a feasible strategy for flexible microwave absorption [6, 7]. In such setup, high content of conductive filler is necessary to build a conductive network and achieve adequate microwave absorptivity [8]. However, with high content of conductive fillers, the fillers tend to aggregate, jeopardizing mechanical property of the absorber [9]. As such, an alternative design of EM absorber involving the replacement of either the filler, the matrix or the microstructure of the composite is highly desired.

Commercial carbon cloth is a simple and widely used flexible substrate. Some impressive achievements of carbon-cloth-based flexible absorber have been recently reported. For example, Che et al. deposited MnO_2_ arrays on a carbon cloth substrate, producing a flexible EM wave absorber that achieved RL of -53.2 dB by modifying phase structure and geometrical shape of the MnO_2_ array [10]. Lately, Che’s group also designed ZnO arrays vertically grown in situ on a flexible conductive carbon cloth substrate, producing EM wave absorbers with superior mechanical properties and enhanced EM attenuation ability [11]. These works have proven that carbon cloth is an ideal skeleton for a flexible EM wave absorber. Unfortunately, commercial carbon cloth is an expensive raw material, hindering commercialization of such inventions.

Cotton, a renewable biomass resource, has a fiber microstructure like carbon cloth in addition to other advantages including superior mechanical strength, low mass, low cost and high natural abundance [12]. More importantly, cotton-derived carbon fibers (CF) do not only inherit these fascinating merits, but also exhibit strong dielectric loss ability [13]. However, dielectric loss of the CF alone is unable to provide sufficiently remarkable microwave absorption for practical uses, making its integration with other components critical in achieving high microwave absorption performance from the synergy between different components [14, 15].

In pursuit of high-performance absorbers, graphene-based magnetic materials are widely studied due to their lightweight feature and their dual energy attenuation mechanisms, namely dielectric and magnetic losses [16]. For example, Fe_3_O_4_–graphene heterostructures exhibited superior RL of − 46.4 dB at thickness of as low as 1.4 mm [17]. Despite their strong microwave absorption abilities, these powder-like materials are lack of flexibility and cannot be used in portable electronics.

In attempt to address the mentioned challenges, we have synthesized a cotton-derived, carbon-fiber-reinforced hybrid paper made of reduced graphene oxide (rGO) and Fe_3_O_4_@C nanowires through facile vacuum filtration assembly. The tuning of the content of CF could modulate optimal thickness of the paper and optimize its impedance matching for different purposes. Finally, the produced paper shows good flexibility, lightweight feature and outstanding microwave absorption, exhibiting a promising prospect for its use in protecting wearable electronics.

## Experimental

### Materials

Cotton was purchased from a local supermarket in Jiangsu province, China. Iron(II) chloride tetrahydrate (FeCl_2_·4H_2_O), nitrilotriacetic acid and isopropyl alcohol were purchased from Nanjing Chemical Reagent Co., Ltd. and used as received. The aqueous solution of dispersed graphene oxide nanosheets (GO, 2 mg mL^−1^) and absolute ethanol were procured from Nanjing Crystal Chemical Co. Ltd.

### Preparation of Carbon Fiber

Typically, 3 g cotton was loaded in a tube furnace and heated at 300 °C for 2 h with a heating rate of 5 °C min^−1^ in air. After carbonization, the obtained bulk CF was first cut into tiny pieces and then dispersed in ethanol to form a homogeneous dispersion with a density of 4 mg mL^−1^ before use.

### Synthesis of Fe-CPNWs

Iron-based coordination polymer nanowires (Fe-CPNWs) were prepared via a facile hydrothermal synthesis. In detail, under vigorous stirring, 3.0 g of FeCl_2_·4H_2_O and 0.9 g of nitrilotriacetic acid were dissolved in 60 mL mixture of isopropyl alcohol and deionized water (DI, 0.3 MΩ cm) with a volume ratio of 1:1. The resulting solution was transferred into a 100-mL Teflon-lined stainless steel autoclave, which was placed in an oven for heating at 180 °C for 9 h. A white solid was formed after heating, which was later washed with DI water and ethanol for several times and then dried at 60 °C for 12 h under vacuum.

### Preparation of Fe_3_O_4_@CNW/rGO/CF Hybrid Films

The as-prepared Fe-CPNWs were dispersed in absolute ethanol and sonicated for 15 min to form a homogeneous dispersion with a density of 1 mg mL^−1^. Then, 12.5 mL of GO solution (2 mg mL^−1^) was mixed with 250 mL of Fe-CPNW-containing ethanol solution, followed by the addition of different volumes of CF-containing ethanol solution (0, 20, 30 and 40 mL). After the solutions were mixed and sonicated for another 30 min, the mixture was left standing for 12 h and then filtered to obtain CF/GO/Fe_3_O_4_@CNW paper. The resultant composite paper was dried under ambient conditions and then carefully removed from the filter paper. Finally, it was placed in a tube furnace, heated with a ramp rate of 2 °C min^−1^ to 600 °C and kept for 3 h in Ar. Upon cooling down to room temperature, the final flexible CF/rGO/Fe_3_O_4_@CNW hybrid paper was obtained. In this article, the obtained hybrid papers with CF contents of 0, 20, 30 and 40 mL are denoted as S0, S1, S2 and S3, respectively.

### Characterization

Microstructures and morphologies were observed using a Hitachi S4800 scanning electron microscope (SEM) and a JEOL JSM-2010 transmission electron microscope (TEM). X-ray powder diffraction (XRD) was performed on a Bruker D8 ADVANCE diffractometer. Raman spectra were recorded using a Renishaw inVia 2000 Raman microscope. X-ray photoelectron spectroscopy (XPS) system with PHI 5000 Versa Probe was used to obtain XPS spectra. Chemical bonding in the samples was analyzed using Fourier-transform infrared (FTIR) spectra, which were recorded by a PerkinElmer 2000 FTIR spectrometer using KBr disks. Thermogravimetry (TG) analysis was conducted using an NETZSCH STA 449F3 thermal gravimetric analyzer with the temperature ranging from 23 to 800 °C in air. The EM absorption parameters were measured via an Agilent PNA N5244A vector network analyzer in the frequency range of 2–18 GHz according to the coaxial-line method. The tested specimens were prepared by homogeneously mixing the paraffin wax with samples at sample-to-wax mass ratio of 2: 8 and then pressing the mixture into toroidal-shaped ring with outer diameter Ф_out_ of 7.00 mm and inner diameter Ф_in_ of 3.04 mm.

## Results and Discussion

The cotton was first treated at a low temperature of 300 °C in air to obtain CF with oxygen-containing functional groups. Figure S1a reveals that the CF has a smooth surface with a diameter of several micrometers. FTIR spectrum of CF is shown in Fig. S1b. The broad peak at around 3400 cm^−1^ is associated with stretching vibrations of OH groups [18]. The peaks at 1705 and 1618 cm^−1^ belong to stretching vibrations of C=O and C=C, respectively [19]. The peak at 1243 cm^−1^ corresponds to stretching vibration of carboxylic anhydride groups [20]. The results confirm that the low-temperature carbonization in air resulted in formation of oxygen-containing functional groups in CF.

Simultaneously, Fe-CPNWs were synthesized using a solvothermal method in a Teflon-lined stainless-steel autoclave. Figure S1c demonstrates that Fe-CPNWs possess well-defined one-dimensional structures with a mean diameter of about 160 nm. The corresponding FTIR spectrum discloses the composition of Fe-CPNWs (Fig. S1d). The peak cantered at 3437 cm^−1^ is ascribed to the coordination between Fe^2+^ and nitrilotriacetic acid [21]. The peak at about 1593 cm ^−1^ belongs to in-plane bending vibration of N–H bond. The peaks at about 1303 and 1028 cm^−1^ are assigned to the stretching vibration and in-plane bending vibration of C-H bond, respectively [22]. GO aqueous solution was homogeneously mixed with Fe-CPNW-containing ethanol solution and CF-containing ethanol solution. In this process, CFs interacted with GO nanosheets through their oxygen-containing functional groups by means of van der Waals forces and hydrogen bonding. Fe^2+^ from Fe-CPNWs also interacted with oxygen-containing functional groups of GO and CF, reinforcing their interactions. From Fig. S2, it is seen that the formed CF/GO/Fe-CPNW composite at the bottom of the beaker is visually homogeneous.

**Fig. 1:**
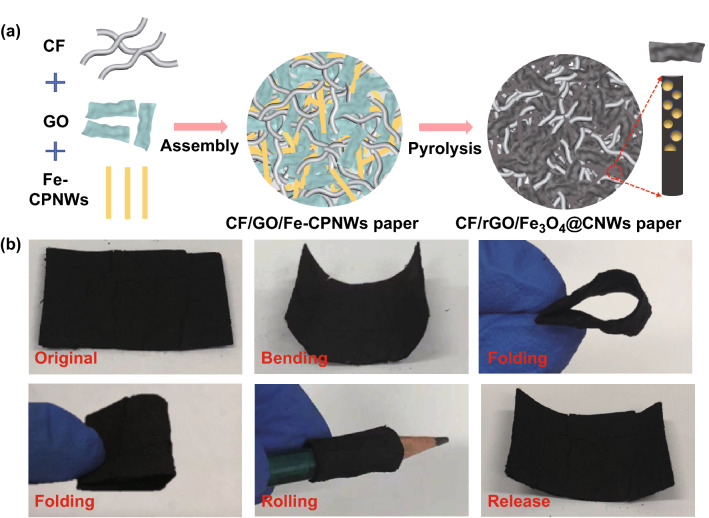
**a** Preparation process of CF/rGO/Fe_3_O_4_@CNW hybrid paper. **b** Digital images showing flexible features of the CF/rGO/Fe_3_O_4_@CNW paper

A free-standing CF/GO/Fe-CPNW paper was produced by vacuum-filtration-induced assembly. After carbonization, Fe-CPNWs were transformed into Fe_3_O_4_@C nanowires (Fe_3_O_4_@CNWs) and elimination of oxygen-containing functional groups converted GO into rGO, leading to the formation of free-standing CF/rGO/Fe_3_O_4_@CNWs hybrid paper. Figure 1a illustrates typical preparation process of the hybrid paper. Figure 1b shows that the resultant hybrid paper has a smooth surface. After bending, rolling and folding, the paper could maintain its structural integrity, exhibiting flexibility and superior mechanical property. It is noted that the control sample S0, without addition of CF, was prone to fracture into small pieces as shown in Fig. S3. Hence, it is inferred that CF played a role in strengthening mechanical property and improving flexibility of the paper.

**Fig. 2:**
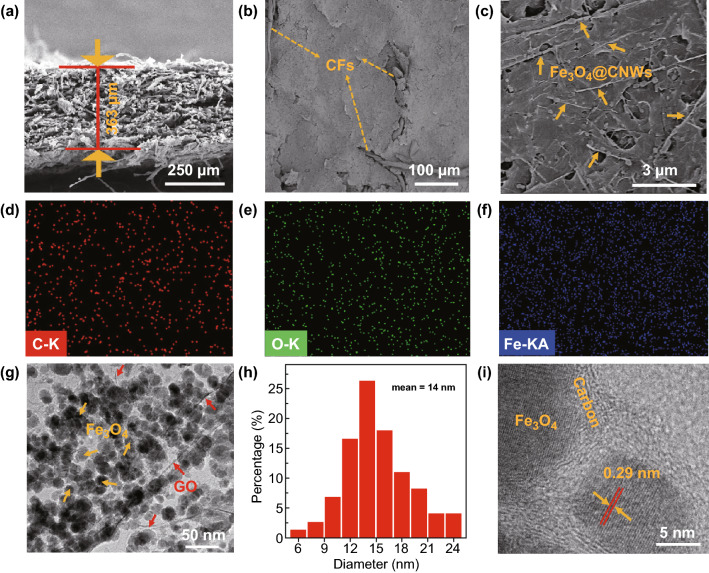
**a** Cross-sectional view and **b–c** top-view FESEM images. **d–f** Elemental mapping of the hybrid paper. **g** TEM image of the hybrid paper. **h** Size distribution of Fe_3_O_4_ nanoparticles. **i** HRTEM image of the hybrid paper

The microstructures of hybrid paper are shown in Fig. 2. The cross-sectional view of the hybrid paper in Fig. 2a exhibits its thickness of about 363 µm, consisting of multiple layers. The thickness of the paper could be tuned between 206 and 449 μm by changing the CF content (Fig. S4). Figure 2b, c demonstrates that the CFs were surrounded by Fe_3_O_4_@CNW and rGO components, forming a relatively flat surface. A close inspection reveals that one-dimensional Fe_3_O_4_@CNWs and rGO nanosheets were tangled with each other. The energy-dispersive X-ray spectroscopy (EDS) spectrum shows strong signals from C, O and Fe elements (Fig. S5). The corresponding elemental mapping reveals that three elements Fe, C and O are evenly distributed throughout the paper (Fig. 2d–f). TEM images (Fig. 2g) reveal numerous Fe_3_O_4_@C nanostructures were intertwined with rGO. As shown in Fig. 2h, the mean diameter of Fe_3_O_4_ nanoparticles (NPs) is about 14 nm. The core–shell structure of Fe_3_O_4_@C could be observed from high-resolution TEM images in Fig. 2i. The lattice fringe spacing of ~ 0.29 nm corresponds to d-spacing of Fe_3_O_4_ {220} crystal planes [23].

**Fig. 3:**
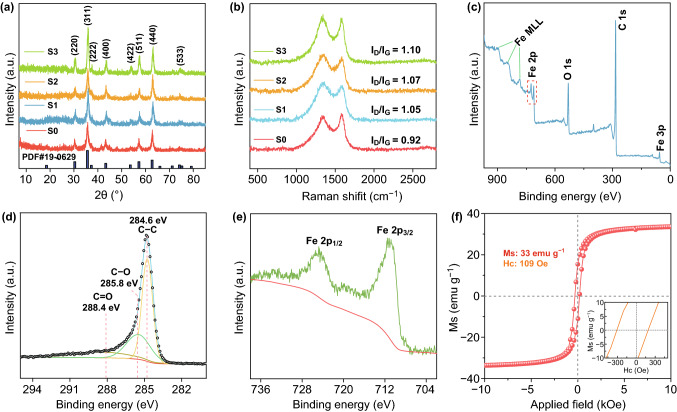
**a** XRD patterns and **b** Raman spectra of all samples. **c** XPS broad survey. **d** C 1 s and **e** Fe 2p spectra of S2. **f** Magnetic hysteresis loop of S2 measured at room temperature

XRD patterns in Fig. 3a reveal the structural information of all samples. The characteristic diffraction peaks of magnetite Fe_3_O_4_ could be found in all samples. The signals at 2θ values of 30.1°, 35.4°, 37.0°, 43.1°, 53.4°, 56.9°, 62.5° and 73.9° can be attributed to (220), (311), (222), (400), (422), (511), (440) and (533) crystal planes of Fe_3_O_4_ (JCPDS No. 19-0629), respectively [24]. It is noted that no characteristic diffraction peaks of carbon could be spotted in the XRD patterns owing to the low loading of rGO and the low crystallinity of CF, which was confirmed by Raman spectra.

In Fig. 3b, all samples show two prominent peaks at about 1357 and 1539 cm^−1^, which correspond to D and G bands of carbon materials, respectively [25]. Generally, the intensity ratio of D band to G band (*I*_D_/*I*_G_) is utilized to assess the degree of disorder in the carbon-based components. Herein, the values of I_D_/I_G_ are 0.92, 1.05, 1.07 and 1.10 for S0, S1, S2 and S3, respectively. With increasing content of CF, *I*_D_/*I*_G_ ratio increases gradually from S0 to S3, indicating increased disorder in the carbon-based components and further confirming the low crystallization degree of CF.

Due to similarity in XRD patterns between magnetite and maghemite [26]. XPS was carried out to provide insights into the elemental composition of the product and the oxidation state of each element. The total survey (Fig. 3c) shows that Fe, C and O elements were present on the surface of composite film. The O elements could be attributed to the residual oxygen-containing functional groups of GO. High-resolution C 1 s spectrum (Fig. 3d) was deconvoluted into three subpeaks corresponding to C–C (284.5 eV), C-O (285.8 eV) and C = O (288.4 eV) [27]. Fe 2p spectrum (Fig. 3e) shows two broad peaks at 710 and 724 eV, which can be ascribed to the ionization of Fe 2p_3/2_ and Fe 2p_1/2_. No satellite peak could be found at around 719.2 eV, which indicates that the as-synthesized Fe_3_O_4_ nanoparticles have high level of purity [28].

TG analysis was carried out in air to evaluate the content of Fe_3_O_4_. Due to oxidation of Fe^2+^, the residue is comprised of Fe_2_O_3_ with mass percentage of about 60 wt% (Fig. S6) on the basis of Eq. 1:

1 $${\text{Fe}}_{3} {\text{O}}_{4} ({\text{wt}}\% ) = {\text{Residue}}({\text{wt}}\% ) \times \frac{{2M_{{{\text{Fe}}_{3} {\text{O}}_{4} }} }}{{3M_{{{\text{Fe}}_{2} {\text{O}}_{3} }} }}$$

Hence, it is inferred that the content of Fe_3_O_4_ in the hybrid paper is ~ 56 wt%. Fe_3_O_4_ could endow the paper with typical magnetic properties as proven in Fig. 3f. The representative sample S2 has a saturation magnetization (*M*_s_) value of 33 emu g^−1^ and a coercive force of 190 Oe. The apparent magnetic behavior would bring about magnetic loss that dissipates EM waves [29].

**Fig. 4:**
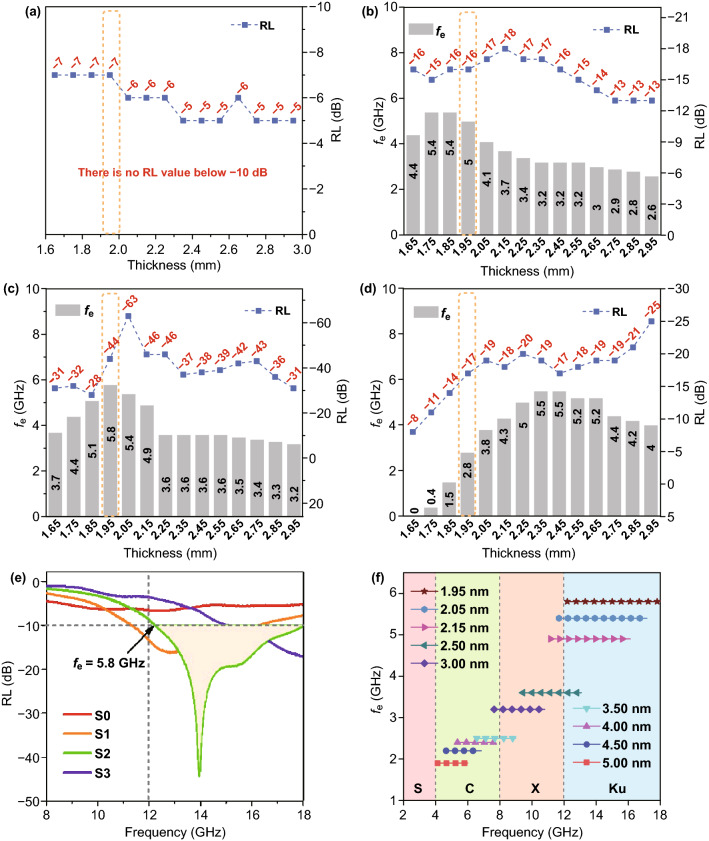
Maximum *RL* and *f*_e_ values of **a** S0, **b** S1, **c** S2 and **d** S3. **e** RL curves of all samples at the thickness of 1.95 mm. **f** Effective absorption frequency bandwidths of S2 with various thicknesses between 1.95 and 5.00 mm

RL values of samples were calculated using measured parameters, namely relative complex permittivity (ε_r_) and relative complex permeability (μ_r_). Based on the transmission line theory, RL can be calculated using Eqs. 2 and 3 [30, 31]:

2 $$Z_{\text{in}} = Z_{0} \sqrt {\frac{{\mu_{r} }}{{\varepsilon_{r} }}} \tanh \left[ {j\left( {\frac{2f\pi t}{c}} \right)\sqrt {\mu_{r} \varepsilon_{r} } } \right]$$

3 $${\text{RL}} = 20\log \left| {\frac{{Z_{\text{in}} - Z_{0} }}{{Z_{\text{in}} + Z_{0} }}} \right|$$

where *Z*_in_ is the input impedance of the absorber, Z_0_ is the impedance of the free space, *f* is the microwave frequency, d is the thickness of absorber and c is the velocity of light. EM wave attenuation at an input wave frequency is considered adequate if RL value is below − 10 dB, representing attenuation of more than 90% of the incident EM energy [32]. The frequency ranges with RL below − 10 dB are defined as effective absorption frequency bandwidth (*f*_e_). Figure 4 shows maximum *RL* (*RL*_max_) and *f*_e_ values of all samples at different thickness. In Fig. 4a, none of the maximum RL values exceeds -10 dB (in magnitude) in S0. When CF was introduced, the microwave absorption performance was remarkably enhanced, as exemplified by S1 that showed a *RL*_max_ of -18 dB and broad *f*_e_ of 5.4 GHz at thicknesses of 2.15 mm and 1.75 mm, respectively (Fig. 4b). With increased CF content, *RL*_max_ of S2 could reach − 63 dB at 2.05 mm and *f*_e_ was as high as 5.8 GHz at 1.95 mm (Fig. 4c). Further increase in CF content has resulted in lower *RL*_max_ of − 25 dB and narrower maximum *f*_e_ of 5.5 GHz in S3, suggesting weakened absorption behavior (Fig. 4d). Among the four samples, S2 demonstrated the best microwave absorption capacity. Notably, the broad *f*_e_ of 5.8 GHz could cover the Ku band almost entirely at thickness of as low as 1.95 mm (Fig. 4e). When the thickness was tuned between 1.95 and 5.00 mm, the accumulated attenuated frequency range could nearly cover the entirety of C, X and Ku bands (Fig. 4f).

**Fig. 5:**
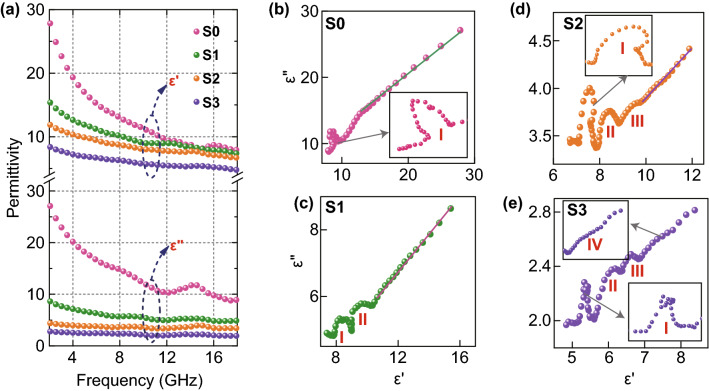
**a** Plot of complex permittivity versus frequency and **b–e** Cole–Cole plots of all samples

EM wave absorption properties are highly associated with the relative complex permittivity (*ε*_*r*_ = *ε′*-*jε″*) and relative complex permeability (*μ*_*r*_ = *μ′*-*jμ″*). In general, the real parts of complex permittivity (*ε′*) and complex permeability (*μ′*) represent the abilities of a material to store electric energy and magnetic energy, respectively, whereas the imaginary parts of the two quantities (*ε″* and *μ″*) represent its abilities for electric energy dissipation and magnetic energy loss, respectively [33, 34]. In Fig. 5a, *ε′* and *ε″* values of all samples decrease with increasing frequency due to typical frequency dispersion behavior, in which polarization lag is aggravated by electric field change at higher frequency [35]. Among them, S0 shows the highest *ε′* (ranging from 27.8 to 7.6) and *ε″* (ranging from 27.1 to 8.9) values. When CF was introduced, both *ε′* and *ε″* values of samples decreased with increasing CF content, possibly due to the reduction in conductive loss. According to the conductive-network model proposed by Cao and his coworker, the conductivity of the composite is significantly influenced by electron hopping between Fe_3_O_4_-rGO layers [36]. When CFs with low degree of graphitization is introduced, the CFs will behave like resistors, hindering interlayer electron hopping and intralayer electron migration, causing the conductive network to be less connected and decreasing conductance of the composite.

According to the Debye theory, *ε*_*r*_ can be expressed by the following equation in terms of *ω* = 2π*f* where *f* is frequency [37]:

4 $$\varepsilon_{r} = \varepsilon^{{\prime }} - j\varepsilon^{{\prime \prime }} = \varepsilon_{\infty } + \frac{{\varepsilon_{s} - \varepsilon_{\infty } }}{{1 + j\omega \tau_{0} }}$$

where *τ*_*0*_, *ε*_*∞*_ and *ε*_*s*_ are relaxation time, dielectric constant at infinite frequency and static dielectric constant, respectively. From Eq. 4, it can be inferred that

5 $$\varepsilon^{{\prime }} = \varepsilon_{\infty } + \frac{{\varepsilon_{s} - \varepsilon_{\infty } }}{{1 + (\omega \tau_{0} )^{2} }}$$

6 $$\varepsilon^{{\prime \prime }} = \frac{{\omega \tau_{0} (\varepsilon_{s} - \varepsilon_{\infty } )}}{{1 + \left( {\omega \tau_{0} } \right)^{2} }}$$

From Eqs. 5 and 6, the relationship between ε′ and ε″ can be further deduced as Eq. 7:

7 $$\left( {\varepsilon^{{\prime }} - \varepsilon_{\infty } } \right)^{2} + \left( {\varepsilon^{{\prime \prime }} } \right)^{2} = \left( {\varepsilon_{s} - \varepsilon_{\infty } } \right)^{2}$$

Therefore, the plot of ε″ versus ε′ would form either one or several semicircles, generally referred to as Cole–Cole semicircles, with each semicircle associated with one relaxation process [38]. Figure 5b–e shows curves of ε″ versus ε′ for each as-prepared hybrid paper. Distinct semicircles demonstrate the occurrence of relaxation processes in EM energy decay that could be ascribed to the presence of functional groups in rGO and multiple heterojunction interfaces including CF/rGO, Fe_3_O_4_@CNW/rGO, Fe_3_O_4_/C and Fe_3_O_4_@CNW/CF. The functional group can work as the dipolar site under the action of EM wave, causing dipolar polarization [39]. Che et al. revealed that the multiple heterojunction would lead to the accumulation and uneven distribution of charges at these interfaces, leading to the production of macroscopic electric moment. This would induce interfacial polarization and decay the incident EM energy [40]. There are one, two, three and four semicircles in S0, S1, S2 and S3, respectively. The increase in the number of semicircles indicates enhancement of the relaxation processes with the increase in CF content. This is attributed to the formation of more heterojunctions within the composite at which more charges could be accumulated with the increase in CF content, enhancing interfacial polarization. An obvious resonance peak of *ε″* at about 14 GHz in S3 further illustrates its strong interfacial polarization. Meanwhile, with the increase in CF content, the slope of *ε″*-*ε′* curves became smoother, indicating decrease in conductivity [41].

**Fig. 6:**
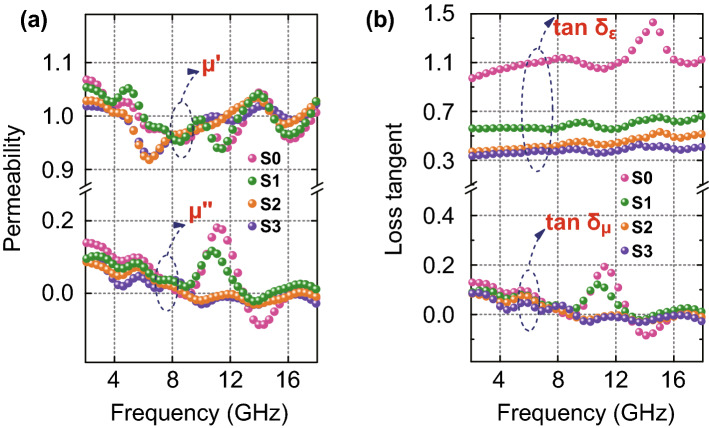
**a** Complex permeability and **b** dielectric and magnetic loss tangents of all samples

From Fig. 6a, it is found that CF content has no significant effect on *μ′* and *μ″* values of the composite. *μ′* and *μ″* vary in the ranges of 0.92–1.07 and -0.08–0.18, respectively. However, the apparent resonance peaks could be found in the plot of *μ″* against frequency, indicating magnetic loss caused by the Fe_3_O_4_. Low-frequency resonance peaks are usually related to natural resonance, whereas high-frequency resonance peaks may correspond to the exchange resonance [42]. Besides, eddy loss is another pathway for magnetic loss. According to previous reports, if the eddy loss contributes to magnetic loss, eddy current coefficient *C*_*0*_ (*C*_*0*_ = *μ″*(*μ*′)^−2^*f*^−1^) will remain constant with variation in frequency [43]. From Fig. S7, *C*_*0*_ of all samples rapidly decreased between the frequencies of 2 GHz and 8 GHz, fluctuated in frequency range of 8–13 GHz and remained constant in the range of 13–18 GHz, suggesting that eddy current loss contributed to microwave attenuation in Ku band.

Dielectric loss tangent (tan *δ*_*ε*_) and magnetic loss tangent (tan *δ*_*μ*_) were also evaluated and plotted as functions of frequency. As shown in Fig. 6b, tan *δ*_*ε*_ values of the composites are greater than their tan *δ*_*μ*_ values, which implies that dielectric loss was the dominant mechanism in EM energy attenuation for the composite [44]. As the CF content increased, the value of tan *δ*_*ε*_ decreased rapidly, while tan *δ*_*μ*_ values did not change significantly, indicating that CF content had no significant influence on magnetic loss. This phenomenon provides opportunity to improve impedance matching, facilitating penetration and attenuation of EM waves.

**Fig. 7:**
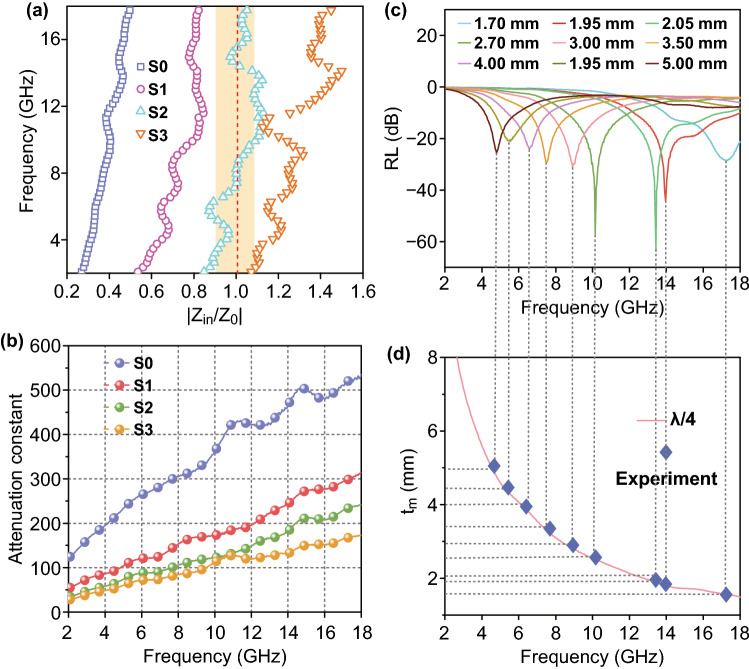
**a** Attenuation constant and **b** |Z_in_/Z_0_| values of all samples. **c** Plots of RL versus microwave frequency at different thicknesses of S2. **d** Relationship between simulated matching thickness *t*_m_ and peak frequency for S2

Figure 7a shows impedance matching values |Z_in_/Z_0_| of all samples. Generally, when the impedance matching ratio |Z_in_/Z_0_| is close to 1, almost all incident EM waves could penetrate the surface of a material with near-zero microwave reflection, presenting an ideal impedance matching [45]. S0 sample has a low |Z_in_/Z_0_| value, resulting in poor impedance matching. However, with the increase in CF content, |Z_in_/Z_0_| values increased significantly. In particular, |Z_in_/Z_0_| of S2 fluctuated around 1, showing excellent impedance matching. When the EM waves penetrate the interior region, the energy should be dissipated as much as possible. The ability of a material to dissipate or attenuate EM energy of a frequency is described by its attenuation constant (α) [46]. As shown in Fig. 7b, α values of all samples exceeded 150 at high frequencies, suggesting strong microwave attenuation capability of these composites. This should be attributed to the synergistic effect of both dielectric and magnetic losses. Hence, the superior microwave absorption capability of S2 should be attributed to the optimal combination of good impedance matching and adequately high attenuation capacity. Table 1 summarizes the EM absorption performance of some representative carbon-based magnetic absorbers in the recent literature [36, 47–54]. It is noteworthy that the strong microwave absorption of S2 at a low filler content of 20% is competitive to those reported EM absorbers.

**Table 1:** EM absorption properties of recently reported carbon-based magnetic absorbers and S2 sample in this work

Sample	Maximum RL value		Filler content	RL≦–10 dB		References
	*D*_m_	*RL*_max_		*D*_m_	*f*_e_	
Fe_3_O_4_/GCs	3.5	− 32	30	3.5	~4.6	[47]
Fe_3_O_4_ clusters-NG	4.1	− 53.6	30	1.8	~5	[36]
WPC/MNPs-80	2.0	− 47.9	33	2.0	4.1	[48]
Co@C nanofiber	2.4	− 40	50	2.4	~2.5	[49]
Porous carbon/Fe_3_O_4_@Fe	2.0	− 49.6	30	2.0	5.0	[50]
Fe@NPC@CF	2.5	− 46.2	25	2.5	5.2	[51]
Nanoporous carbon	2.0	− 42.4	70	2.0	1.76	[52]
FeNi_3_/N-GN	1.45	− 57.2	50	1.94	4.2	[53]
Fe_3_O_4_@NPC	3.0	− 65.5	40	3.0	4.5	[54]
S2	2.05	− 63	20	1.95	5.8	This work

To understand the relationship between thickness and peak frequency (*i.e.,* microwave frequency for which maximum RL was recorded for a given absorber thickness), the curves of RL versus frequency for S2 at different thicknesses are plotted in Fig. 7c. It is obvious that the peak shifts toward lower frequencies with increasing matching thickness, which can be explained by the quarter-wavelength cancelation model expressed by Eq. 8 [55, 56]:

8 $$t_{m} = n\lambda /4 = nc/(4f_{m} \sqrt {\mu_{r} \varepsilon_{r} } )\quad (n = 1,3,5 \ldots )$$

where *c* is the speed of EM waves in free space, t_m_ is matching thickness and *f*_m_ is matching (or peak) frequency. When the values of *t*_m_ and *f*_m_ satisfy this equation, EM waves reflected at air–absorber interface and EM waves reflected at absorber–device interface would be 180° out of phase, causing them to be canceled out due to destructive interference and leading to the high RL value [57]. In this case, peak frequencies and their corresponding matching thicknesses are proven to be consistent with the simulated values deduced from Eq. 8 (as shown in Fig. 7d). Hence, the quarter-wavelength cancelation model is vital to predict the absorption frequency and thickness of maximum RL for an EM absorber [58].

**Fig. 8:**
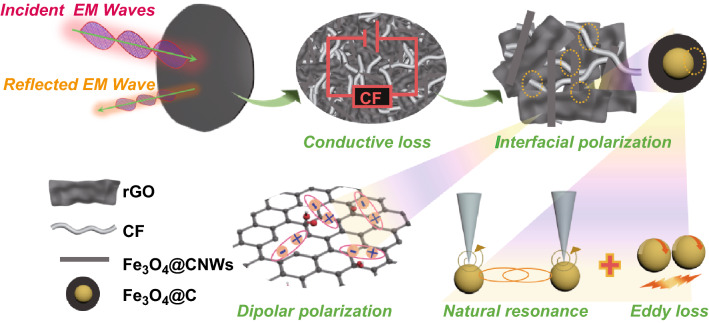
Possible microwave absorption mechanisms for CF/rGO/Fe_3_O_4_@CNW hybrid paper

Based on the above discussion, the mechanisms of microwave absorption in CF/rGO/Fe_3_O_4_@CNW hybrid paper can be summarized in Fig. 8. Firstly, a maximized fraction of incoming EM waves (particularly microwaves) penetrated the air–absorber interface with a good impedance matching. Then, energy of the penetrated EM waves decayed through various mechanisms, expounded as follows:

- Conductive loss. In the composite, rGO nanosheets, Fe_3_O_4_@CNWs and CF formed a conductive network with finite resistance. As the incident EM wave propagated through the resistive conductive network, the induced microcurrent was rapidly attenuated in the resistive network and converted to thermal energy, leading to the decaying of penetrated EM energy.
- Polarization loss. Various phase boundaries in Fe_3_O_4_@CNW-rGO-CF could accumulate the charges, inducing interfacial polarization. Meanwhile, oxygen-containing functional group residues and defects in rGO could work as dipolar centers, causing dipolar polarization. Two kinds of polarization process could attenuate the incident EM wave.
- Magnetic loss. Natural resonance, exchange resonance and eddy loss resulting from the magnetic properties of the Fe_3_O_4_ core in Fe_3_O_4_@CNW contribute to the magnetic energy loss of the hybrid paper.

## Conclusion

In summary, a lightweight and flexible CF/rGO/Fe_3_O_4_@CNW hybrid paper has been successfully fabricated through a vacuum filtration assembly process. Therein, carbon fibers (CFs) work as flexible backbone, tightly wrapped by the other dielectric and magnetic components including reduced graphene oxide (rGO) and Fe_3_O_4_@C nanowires (Fe_3_O_4_@CNWs). The strongest microwave absorption intensity of -63 dB can be achieved at a thickness of 2.05 mm, while the largest effective absorption bandwidth *f*_*e*_ is 5.8 GHz with a thickness as low as 1.95 mm. The fabrication approach for such a hybrid EM absorption paper with flexibility and lightweight features provides a promising way for the future development of lightweight and effective microwave absorbers.

## Electronic supplementary material

Below is the link to the electronic supplementary material.

Supplementary material 1 (PDF 467 kb)
