# Supplementary material for: A Flexible and Lightweight Biomass-Reinforced Microwave Absorber
Source: Nanomicro Lett. 2020 Jun 11;12:125. doi: 10.1007/s40820-020-00461-x (PMC7770825; doi:10.1007/s40820-020-00461-x)
Supplement: Supplementary file 1 — Supplementary material 1 (PDF 467 kb) [file 40820_2020_461_MOESM1_ESM.pdf]

Supporting Information for

A Flexible and Lightweight Biomass-Reinforced Microwave Absorber

Yan Cheng[1, ‡], Justin Zhu Yeow Seow[2, 3, ‡], Huanqin Zhao[1], Zhichuan J. Xu[2, 3, *], Guangbin Ji[1, *]

[1]College of Materials Science and Technology, Nanjing University of Aeronautics and Astronautics, Nanjing 210016, People's Republic of China

[2]School of Materials Sciences and Engineering, Nanyang Technological University, 50 Nanyang Avenue, Singapore 639798

[3]Singapore-HUJ Alliance for Research and Enterprise, NEW-CREATE Phase II,

Campus for Research Excellence and Technological Enterprise (CREATE), Singapore 138602

‡Yan Cheng and Justin Zhu Yeow Seow contributed equally to this work

*Corresponding authors. E-mail: gbji@nuaa.edu.cn (Guangbin Ji),

xuzc@ntu.edu.sg (Zhichuan J. Xu)

Supplementary Figures

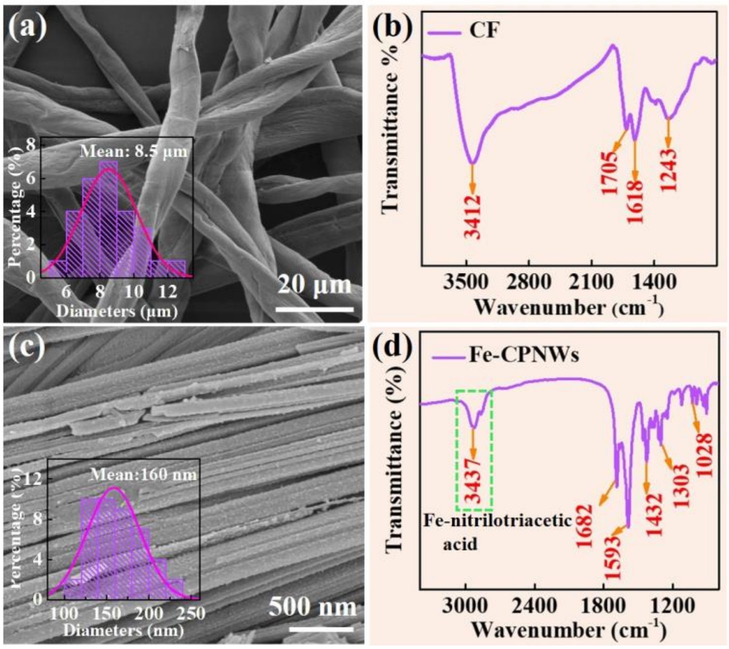

Fig. S1 (**a**) SEM images of cotton-derived CFs, (**b**) FT-IR spectrum of CFs, (**c**) SEM image of Fe-CPNWs, and (**d**) diameter distribution of Fe-CPNWs

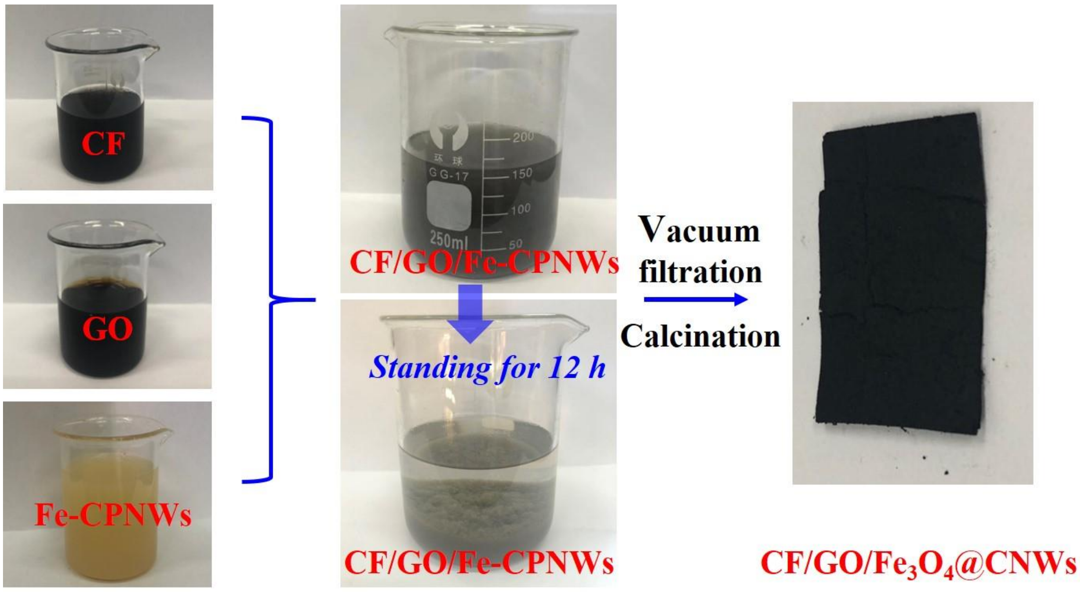

Fig. S2 Assembly of CF/GO/Fe-CPNWs from CF-containing ethanol, GO solution and Fe-CPNW-containing ethanol

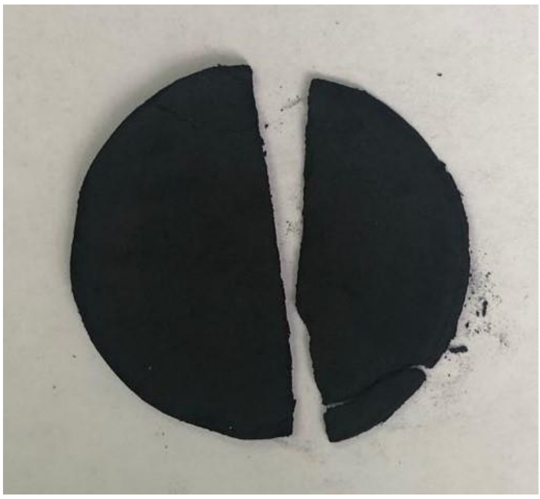

Fig. S3 Digital image of S0

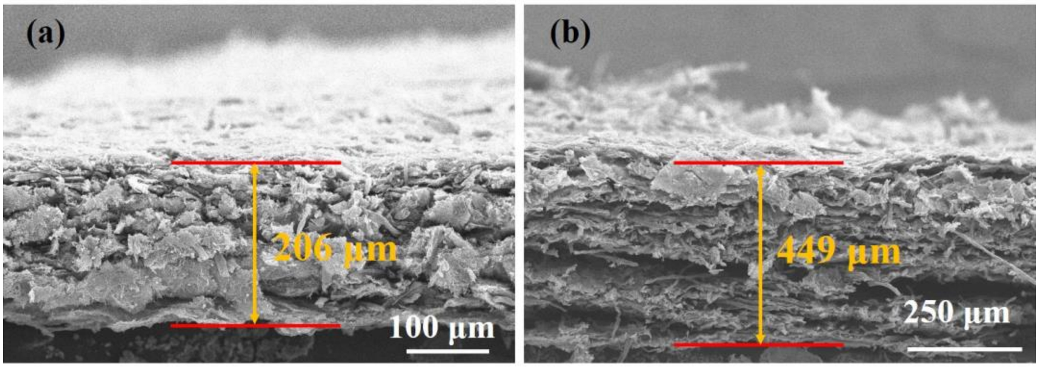

Fig. S4 Cross-sectional SEM images of (**a**) S1 and (**b**) S2

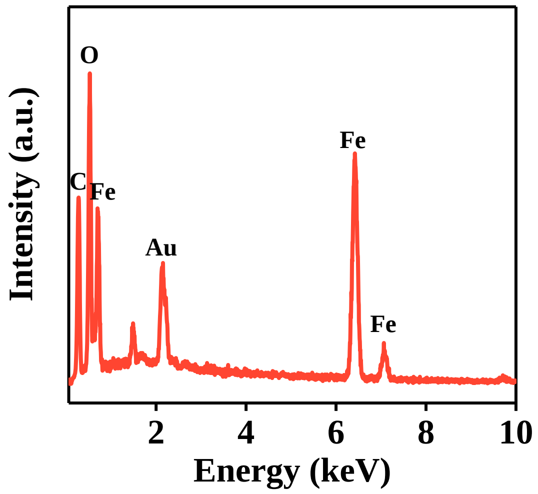

Fig. S5 EDS spectrum of S2

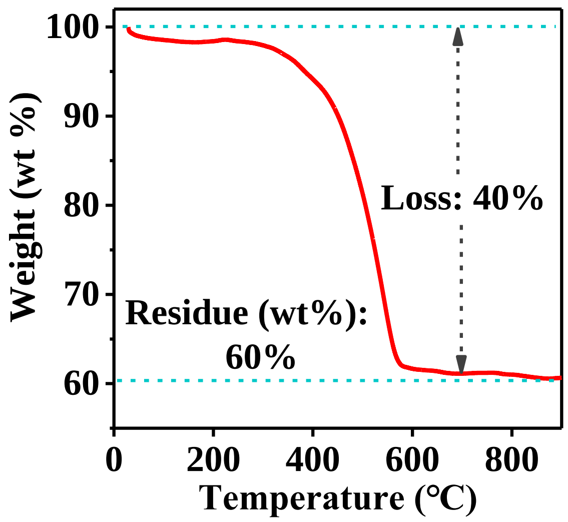

Fig. S6 TG curve of S2

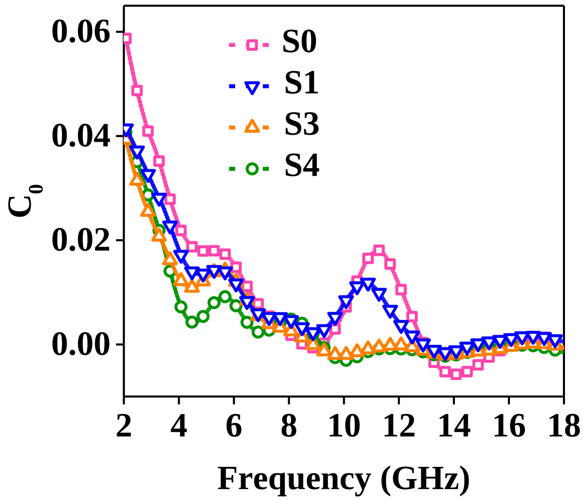

Fig. S7 The eddy current coefficient C_0 of all samples
